# Treatment options for resectable hypopharyngeal squamous cell carcinoma: A systematic review and meta-analysis of randomized controlled trials

**DOI:** 10.1371/journal.pone.0277460

**Published:** 2022-11-29

**Authors:** Smriti Panda, Pirabu Sakthivel, Kurinchi S. Gurusamy, Atul Sharma, Alok Thakar

**Affiliations:** 1 Department of Otorhinolaryngology and Head and Neck Surgery, All India Institute of Medical Sciences, New Delhi, India; 2 Division of Surgery and Interventional Science, UCL, London, United Kingdom; 3 Department of Therapy, I.M. Sechenov First Moscow State Medical University, Moscow, Russian Federation; 4 Department of Medical Oncology, All India Institute of Medical Sciences, New Delhi, India; University Putra Malaysia, MALAYSIA

## Abstract

**Background:**

There is uncertainty in the treatment options for resectable hypopharyngeal squamous cell carcinoma.

**Methods:**

A systematic review of randomised controlled trials (RCTs) was performed. Cochrane Central Register of Controlled Trials (CENTRAL) (The Cochrane Library), MEDLINE, EMBASE, Science Citation Index, and Conference Proceedings databases and trial registries were searched until November 2020 for randomized controlled trials performed on resectable hypopharyngeal squamous cell carcinoma. Two systematic review authors independently identified studies and extracted data. The primary outcomes evaluated were overall survival, disease-free survival, any recurrence, local recurrence, loco-regional recurrence, distal recurrence and laryngectomy-free survival. The secondary outcomes were response rates following neoadjuvant treatment and comparison of treatment-related toxicity. Assessment of risk of bias was performed for the selected studies using Cochrane’s tool for assessing risk of bias. The studies were evaluated for the quality of evidence using GRADE (Grading of Recommendations, Assessment, Development and Evaluations). Risk ratios (RR), rate ratios, and hazard ratios (HR) were calculated along with 95% confidence intervals (95% CI). The Meta-analysis was performed using a random-effects model.

**Results:**

Five RCTs met the inclusion criteria for this review. The risk of bias was unclear or high for the trials. Non-organ preservation(n = 140) versus organ preservation (n = 144) (two trials): no statistically significant difference could be identified for any of the primary outcomes. Concurrent chemoradiotherapy (n = 37) versus sequential chemotherapy followed by radiotherapy (n = 34) (one trial): no statistically significant difference was noted between the two treatment arms for overall survival, disease-free survival and loco-regional recurrence. Laryngectomy-free survival was found to be superior in concurrent chemoradiotherapy arm (HR:0.28, 95% CI 0.13, 0.57). Induction chemotherapy followed by concurrent chemoradiotherapy (n = 53) versus induction chemotherapy followed by radiotherapy (n = 60) (one trial): no statistically significant difference was noted between the treatment arms for overall survival, disease-free survival and laryngectomy-free survival. Preoperative radiotherapy (n = 24) versus postoperative radiotherapy (n = 23) (one trial): overall survival was found to be better in the postoperative radiotherapy arm (HR:2.44, 95% CI1.18, 5.03). No statistically significant difference was noted in terms of treatment-related toxicity.

**Conclusions:**

There are considerable uncertainties in the management of resectable hypopharyngeal cancer.

**Trail registration:**

PROSPERO registration: CRD42019155613.

## Introduction

Hypopharyngeal cancer includes cancers of the pyriform sinus, post cricoid area and posterior pharyngeal wall. Hypopharyngeal cancers are rare and account for less than 0.5% of all cancers and 3–5% of head and neck cancers [1]. They have a large geographical variation—being relatively rare in Eastern Asia, Africa and Northern Europe (incidence under 0.5:100, 000) and more common in India, Brazil, Central and Western Europe (incidence of 2.5:100, 000). The incidence in India and France are substantially higher at 8–15 cases per 100, 000 population in males [1]. It is an aggressive cancer and reported to have the highest mortality rates among head and neck cancers [1, 2]. Late clinical presentation is typical, with 70%–90% of patients presenting at Stage III or IV disease and is at least in part attributable to the anatomy and location of the hypopharynx [3]. The estimated 5-year overall survival rate for treated stage III and IV hypopharyngeal cancer patients varies between approximately 15% and 40%, depending on tumor-related factors, patient-related factors, and treatment approaches [4].

Treatments applied to cancer hypopharynx have included surgery, radiotherapy (RT), concurrent chemoradiotherapy(CRT), or a combination of these. Total laryngopharyngectomy with postoperative radiotherapy, despite being organ ablating and severely disabling, was the treatment of choice for locally advanced cancers and was considered the standard of care till the 1990s. Larynx removal was advocated because of its anatomical proximity and also risks of aspiration if it was retained, and non-surgical treatment with radiotherapy was noted to be associated with poorer cure rates [5]. Current practice, however, aims for both oncologic cure and laryngeal preservation and is enabled by great sophistication in assessment by radiology and greater efficacy of non-surgical treatments by the addition of chemotherapy to radiotherapy. Laryngeal preservation may be undertaken by surgical or non-surgical methods. In the context of hypopharyngeal cancers however, surgical organ preservation by a partial laryngopharyngectomy is occasionally feasible in very select patients with T1-T2 cancers of the upper hypopharynx and also good pulmonary reserve [6], and organ preservation/ laryngeal preservation is only feasible by non-surgical means in the vast majority of instances.

Non-surgical organ preservation has been explored for laryngeal cancers and for hypopharyngeal cancers by induction chemotherapy (IC) and concurrent chemoradiotherapy (CRT) (Veterans Affair, EORTC 24891, and RTOG 91–11 trials) [7–9] and is now recommended practice for moderately advanced (T3) laryngeal cancers [10]. In comparison to laryngeal cancers, hypopharyngeal cancers have distinctly different biology, a higher rate of regional and distant metastases, poor ability to salvage late recurrence with surgery, and poorer overall prognosis [11]. Extrapolation of the results of the trials conducted for laryngeal squamous cell carcinoma (SCC) and subgroup analyses of multi-site head-and-neck squamous cell carcinoma trials to the hypopharynx is therefore not appropriate. Whether, and in which situations, the attempt towards organ preservation is appropriate with regards to oncological and survival outcomes is an open and unanswered question [12].

Currently available published systematic reviews on hypopharyngeal cancer include retrospective data along with prospective randomized controlled trial data and are limited by the risk of bias inherent in retrospective data [2, 13], i.e., the risk of bias in the studies were not considered while arriving at conclusions. This current meta-analysis is as per the guidelines for a Cochrane review and restricts itself to the available prospective randomized controlled trials comparing the efficacy of organ preservation techniques (IC, CRT and RT) and non-organ preservation techniques (surgery) for management of resectable hypopharyngeal cancers.

## Materials and methods

The detailed protocol has been registered in PROSPERO (CRD42019155613) [14]. PRISMA (Preferred Reporting Items for Systematic Reviews and Meta-analyses) 2009 guidance was used to conduct and report this systematic review [15].

### Criteria for selection

Only randomized controlled trials (RCT) performed in patients with resectable hypopharyngeal cancers were considered for inclusion. Studies involving patients with non-squamous histology, carcinoma-in-situ and unresectable hypopharyngeal cancers were excluded. Comparisons involving total laryngopharyngectomy followed by adjuvant radiotherapy and/or organ-preservation modalities like radiotherapy and chemoradiotherapy (concurrent and sequential) were included in this review. The primary outcomes evaluated were overall survival, disease-free survival, any recurrence, local recurrence, loco-regional recurrence, distal recurrence and laryngectomy-free survival. The secondary outcomes were response rates following induction chemotherapy and comparison of treatment-related toxicity. There were no language restrictions.

### Databases searched

Cochrane Central Register of Controlled Trials (CENTRAL) (The Cochrane Library), MEDLINE, EMBASE, Science Citation Index, and Conference Proceedings databases were searched until 21 November 2020. We also searched the clinical trial registries: Clinicaltrials.gov (http://www.ClinicalTrials.gov/) and The World Health Organization International Clinical Trials Registry Platform (www.who.int/trialsearch) on 21 November 2020. The detailed search strategy is available in **S1 Table**.

### Study selection

Screening of retrieved records (after removal of duplicates) was performed independently by SP and PS. Full texts were retrieved for any records identified as relevant by the screening of titles and abstracts. The final study selection was performed independently by SP and PS based on full texts. Any discrepancy in screening and final study selection was resolved by KG and AT.

### Assessment of risk of bias

Assessment of risk of bias was performed for the selected studies using Cochrane’s tool for assessing risk of bias [16]. The risk of bias was evaluated based on the following domains: sequence generation of randomization, allocation concealment, blinding, incomplete outcome data, selective outcome reporting, and other sources of bias [16]. The risk of bias assessment was performed independently by SP and PS. Any discrepancy was arbitrated by KG and AT.

### Data extraction

Data extraction from selected studies was conducted independently by SP and PS using a predefined Microsoft Excel-based data extraction form designed by KG and piloted by SP and PS. Discrepancies in the data extracted were resolved by AT and KG. Data collected from the studies were: country of origin, number of centres involved, patient selection criteria, description of the intervention in each arm, the sample size in each arm with post-randomization drop-out, risk of bias and outcome data.

### Statistical analysis

The statistical analysis was performed using Revman 5.4.1 [17]. Due to the clinical heterogeneity identified between the studies, the meta-analysis was performed using a random-effects model where applicable. For the time to event outcomes (overall survival, disease-free survival and laryngectomy-free survival), the logarithm of hazard-ratio and standard error were calculated using methods described by Parmar et al [18]; the meta-analysis was performed using the generic-inverse variance method. For the binary outcomes (any recurrence, local recurrence, loco-regional recurrence, regional recurrence and distal recurrence), risk ratios were calculated, and the meta-analysis was performed using the Mantel-Haenszel method where applicable. For the treatment-related complications and toxicities, the logarithm of rate ratio and standard error were calculated; the meta-analysis was performed using the generic inverse method where applicable.

### Heterogeneity

We explored the clinical differences between the studies by comparing the inclusion and exclusion criteria of the participants, the way the intervention and control treatments were delivered, and the outcomes measured. We assessed the methodological differences by comparing the risk of bias in the studies.

We assessed the statistical heterogeneity between the studies using I^2^. We interpreted I^2^ using the guidance available from the Cochrane handbook.

In the presence of statistical heterogeneity, we planned to perform various subgroup analyses (hypopharyngeal subsite, T classification, N classification) but did not perform these because of the paucity of data.

### Reporting bias

We planned to explore reporting bias using funnel plot visual asymmetry and statistical tests for funnel plot asymmetry but did not perform these because of the few trials in this review.

### Grading of certainty of evidence

The studies were evaluated for the certainty of evidence using GRADE (Grading of Recommendations, Assessment, Development and Evaluations). The overall certainty was graded as very low, low, moderate or high. The domains considered for calculating the overall certainty were: risk of bias, imprecision, inconsistency, indirectness and publication bias [19].

### Sample size calculations

The sample size was calculated to assess the adequacy of sample size in existing trials and to direct future randomized controlled trials using the software Power and Sample size PS version 3.1.2.

## Results

### Description of studies

**Fig 1:**
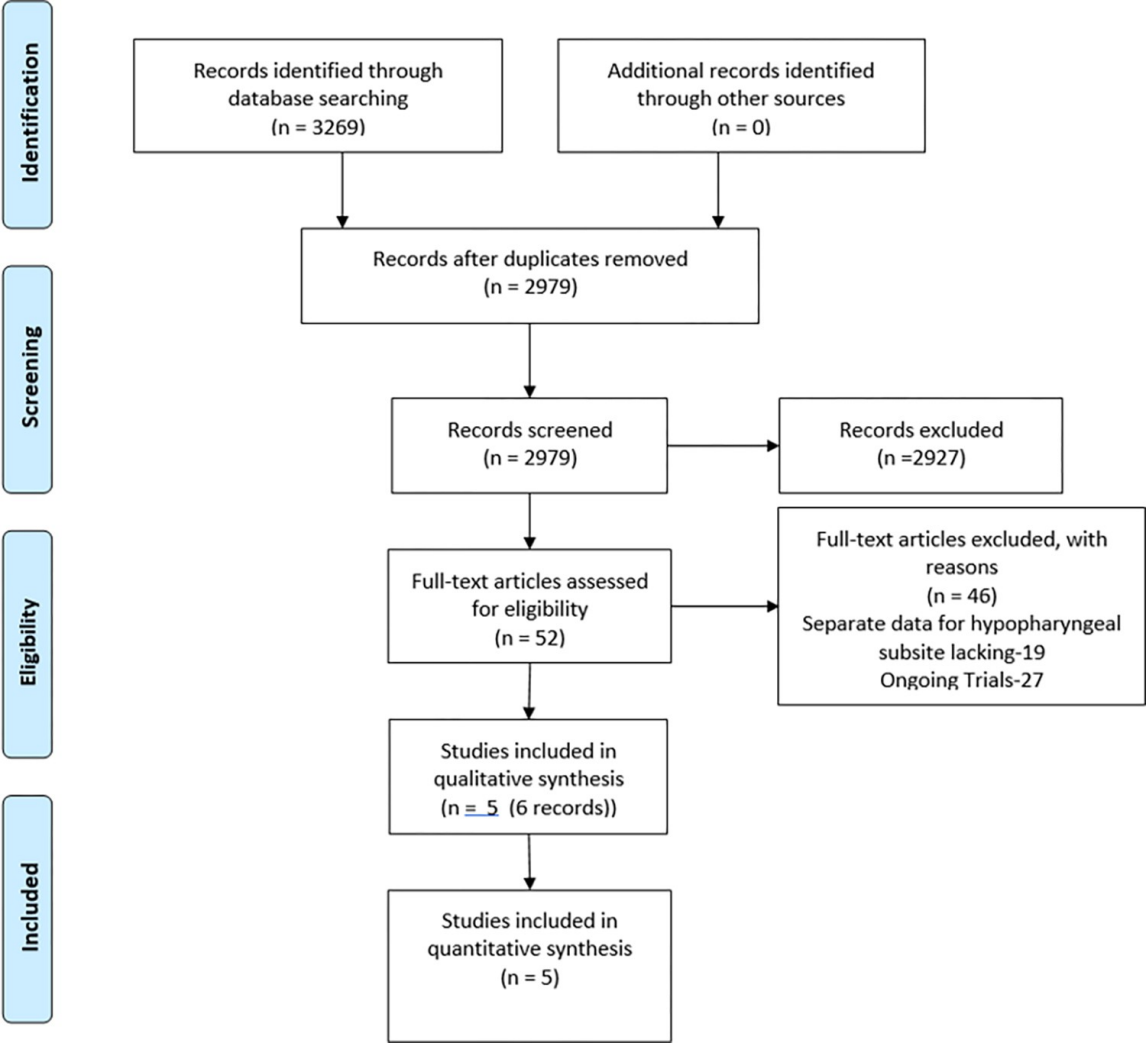
PRISMA flow-chart.

We identified a total of 3269 references. The PRISMA (Preferred Reporting Items for Systematic Reviews and Meta-Analyses) flow diagram for study selection is depicted in **Fig 1** and the PRISMA checklist for this manuscript is available as **S1 Fig**.

**Fig 2:**
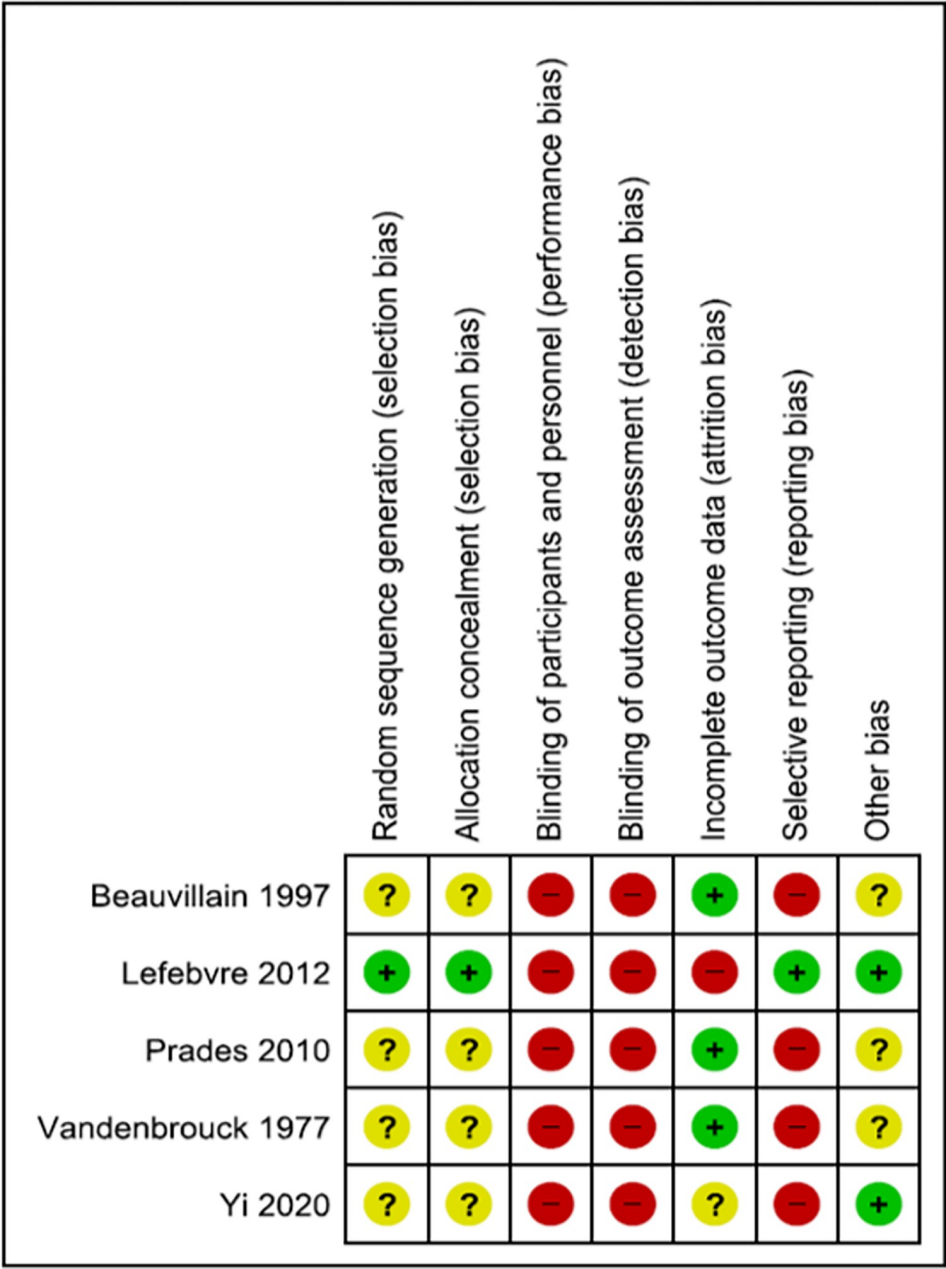
Figure demonstrating risk of bias (ROB).

Of these 3269 references, 5 randomized controlled trials (6 references) were eventually included for the meta-analysis. The risk of bias (ROB) of the trials included has been represented in **Fig 2**. The descriptive details of the trials have been summarized in **Table 1** [20–24]. There were a total of 4 comparisons in these 5 trials.

**Table 1:** Characteristics of included studies.

Study	Inclusion Criteria	Subsite	Intervention I	Intervention II	Sample Size (Intervention I: Intervention II)	Mean Follow up (years)
***Non-organ preservation versus organ preservation***						
Beauvillain 1997 [20]	T3, T4, N0-N3, resectable hypopharyngeal SCC	PFS-90	3 cycles 2-drug induction chemotherapy followed by surgery (day 45) and postoperative radiotherapy	3 cycles 2-drug induction chemotherapy followed by radiotherapy on day 45	46:44	7.7
Lefebvre 2012 [21]	Pyriform sinus or hypopharyngeal aspect of aryepiglottic folds Classified as T2 (AJCC, 1987)—as T2, T3, T4 N0, N1, N2a or N2b neck involvement N2c included till 1990	PFS-152 AEF-42	Immediate surgery with postoperative radiotherapy 50–70 Gy	2-drug induction chemotherapy. CR after three cycles → radiotherapy, less than CR → surgery	94:100	10.5
***Concurrent chemoradiotherapy versus sequential chemotherapy followed by radiotherapy***						
Prades 2010 [22]	Previously untreated T3 pyriform sinus SCC with fixed cords	T3 PFS	Pretreatment neck dissection followed by concurrent chemoradiotherapy	Pretreatment neck dissection followed by 2-drug induction chemotherapy. Participants with complete response or 80% partial response received radiotherapy and participants with <80% partial response received surgery	37:34	2
***Preoperative radiotherapy versus postoperative radiotherapy***						
Vandenbrouck 1977 [23]	Tumors confined to the mucosa of the pyriform sinus, aryepiglottic fold, and arytenoid area	PFS-28 Marginal Zone-7 Pharyngoesophageal junction-7 AEF-3 Hypopharynx unspecified-2	Preoperative radiotherapy followed by surgery	Surgery followed by postoperative radiotherapy	24:23	5
***Induction chemotherapy followed by concurrent chemoradiotherapy (IC-CRT) versus induction chemotherapy followed by radiotherapy (IC-RT)***						
Yi 2020 [24]	Stage III-IV A-B hypopharyngeal cancer	Unknown	3-drug induction chemotherapy followed by concurrent chemoradiotherapy (surgery for primary not reaching large partial response)	3-drug induction chemotherapy followed by radiotherapy (surgery for primary not reaching large partial response)	53:60	3.4

Abbreviations: SCC—Squamous cell carcinoma, PFS—Pyriform sinus, AEF—Aryepiglottic foldT—Tumor stage, N—Nodal stage

### Effect estimates

**Fig 3:**
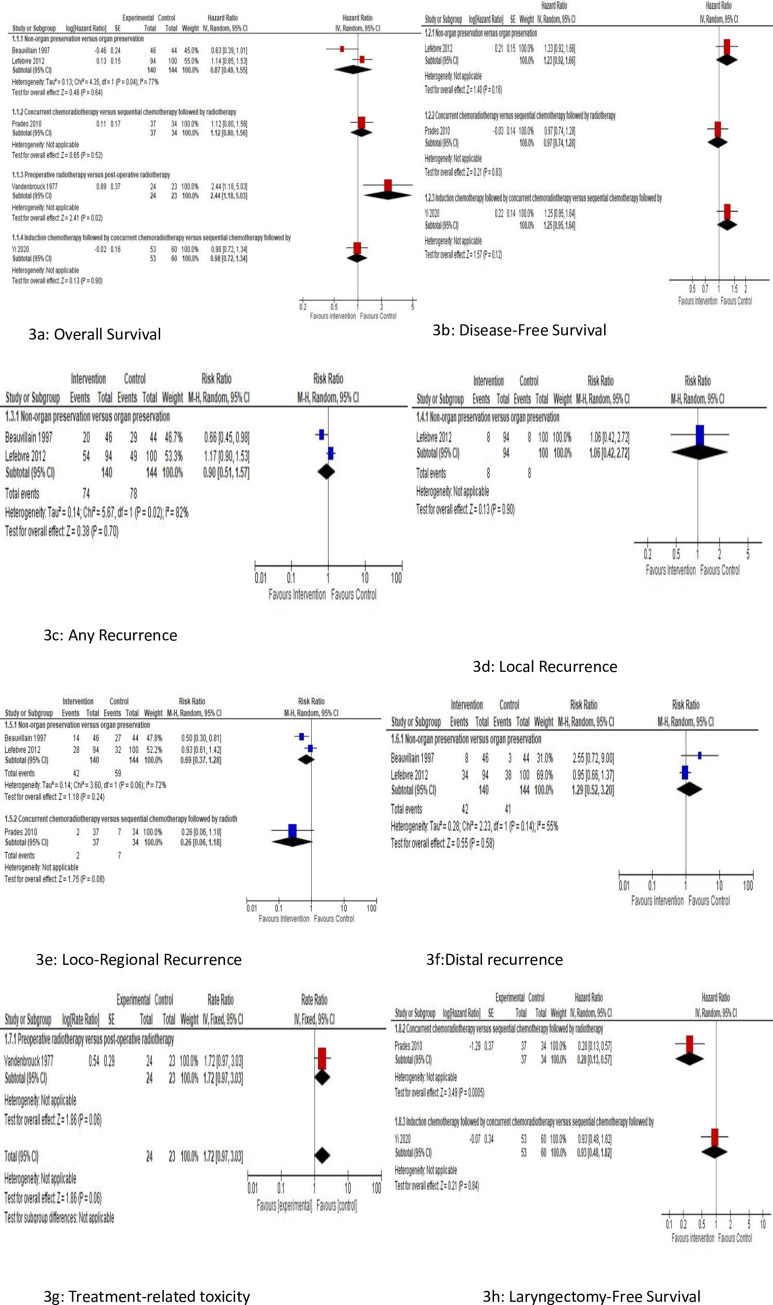
Forest plots for various comparisons and outcomes. a: Overall Survival. b: Disease-Free Survival. c: Any Recurrence. d: Local Recurrence. e: Loco-Regional Recurrence. f: Distal Recurrence. g: Treatment-Related Toxicity. h: Laryngectomy-Free Survival.

The forest plots for each pairwise comparison are provided in **Fig 3**. For response rates and treatment-related toxicity, it was not possible to perform a quantitative analysis. Effect estimates could only be calculated for laryngectomy-free survival for two out of four comparisons. Narrative summaries of the outcomes where quantitative analysis could not be performed are presented in **Table 2** (response rates and laryngectomy-free survival) and **Table 3** (treatment related-toxicity).

**Table 2:** Summary of treatment-related toxicity.

Study	Intervention I	Intervention II	Toxicity: Intervention I*	Toxicity: Intervention II*	Reason why quantitative analysis was not performed
***Non-organ preservation versus organ preservation***					
Beauvillain 1997 [20]	2-drug induction chemotherapy followed by surgery	2-drug induction chemotherapy followed by radiotherapy	24/46	23/44	Inadequately reported: only toxicities related to chemotherapy were reported
Lefebvre 2012 [21]	Immediate surgery followed by radiotherapy	2-drug induction chemotherapy followed by radiotherapy in responders and surgery in non-responders	2/94	15/100	Inadequately reported: only toxicities requiring cessation of treatment were reported
***Concurrent chemoradiotherapy versus sequential chemotherapy followed by radiotherapy***					
Prades 2010 [22]	Pre-treatment neck dissection followed by concurrent chemoradiotherapy	Pre-treatment neck dissection followed by 2-drug induction chemotherapy followed by radiotherapy in responders and surgery in non-responders	50/37	49/34	Inadequately reported: surgical complications were not mentioned suggesting that bias
Preoperative radiotherapy versus postoperative radiotherapy					
Vandenbrouck 1977 [23]	Preoperative radiotherapy followed by surgery	Surgery followed by post-operative radiotherapy	25/24	14/23	Rate ratio calculated and reported
Induction chemotherapy followed by concurrent chemoradiotherapy (IC-CRT) versus induction chemotherapy followed by radiotherapy (IC-RT)					
Yi 2020 [24]	3-drug induction chemotherapy followed by concurrent chemoradiotherapy (surgery for primary not reaching large partial response)	3-drug induction chemotherapy followed by radiotherapy (surgery for primary not reaching large partial response)	40.78%**	5.08%**	Inadequately reported: The denominator was not reported clearly–so number of events could not be calculated

*- Number of Events/number of participants

**- p<0.001

**Table 3:** Response assessment following induction chemotherapy and larynx preservation rates.

Study	Response assessment	Response definition	Response rate	Laryngeal preservation rate
Non-organ preservation versus organ preservation				
Beauvillain 1997 [20]	Oropharyngeal fibreoptic evaluation under general anesthesia between day 35 and 45 of induction chemotherapy	Not reported	Intervention I: Complete response: 3 (tumor), 7 (node); partial response: - 28 (tumor), 11 (node); stabilisation: 15 (tumor), 15 (node), progression: 0 Intervention II: Complete response: 10 (tumor), 6 (node); partial response: 25 (tumor), 16 (node); stable: 8 (tumor), 7 (node); progression:1 (tumor), 1 (node)	Not reported
Lefebvre 2012 [21]	Endoscopy before every cycle of induction chemotherapy and 2 weeks after the previous cycle. CT scan was recommended but not mandatory.	Complete response: complete resolution of the primary tumor with return of laryngeal mobility Partial response: decrease in primary tumor area greater than or equal to 50% Progression: increase greater than 25%	Reported only for 97 participants in the organ preservation group Complete response: 52; partial response: 31; stable: 13; progression: 1	Organ preservation group: 28% at 3 years, 15% at 5 years, and 8.7% at 10 years Non-organ preservation group: not applicable
**Concurrent chemoradiotherapy versus sequential chemotherapy followed by radiotherapy**				
Prades 2010 [22]	Endoscopic evaluation and CT imaging at 3 weeks after completion of induction chemotherapy	Not reported		Concurrent chemoradiotherapy- 92%, Sequential chemotherapy followed by radiotherapy-67.65% at 2 years
**Preoperative radiotherapy versus postoperative radiotherapy**				
Vandenbrouck 1977 [23]	Not reported			Not reported
**Induction chemotherapy followed by concurrent chemoradiotherapy (IC-CRT) versus induction chemotherapy followed by radiotherapy (IC-RT)**				
Yi 2020 [24]	Not reported			Induction chemotherapy followed by concurrent chemoradiotherapy:86.3% Induction chemotherapy followed by radiotherapy:85.4% at 3 years

### Non-organ preservation versus organ preservation

Under this comparison, there was a total of 284 patients (non-organ preservation:140, organ preservation: 144) in two trials [20, 21]. Data pertaining to the primary objectives which could be extracted were overall survival [20, 21], loco-regional and distal recurrence [20, 21], disease-free survival [21], and local recurrence [21]. The pooled HR (Hazard Ratio) of death, distal recurrence and any recurrence did not show any statistically significant difference between the two modalities (overall survival: HR 0.87, 95% CI 0.49, 1.55; distal recurrence: HR 1.29, 95% CI 0.52, 3.20; any recurrence: HR 0.90, 95% CI 0.51, 1.57). There was also no statistically significant difference between the two interventions for local recurrence (HR 1.06, 95% CI 0.42, 2.72), locoregional recurrence (HR 0.69, 95% CI 0.37,1.28), and disease-free survival (HR 1.23, 95% CI 0.92, 1.66) based on the trial by Lefebvre et al [21].

### Concurrent chemoradiotherapy versus sequential chemotherapy followed by radiotherapy

Only one trial was identified comparing these two interventions (71 patients; Concurrent chemoradiotherapy-37 versus Sequential chemoradiotherapy- 34) [22]. The primary outcomes available were overall survival, disease-free survival, loco-regional recurrence, and laryngectomy- free survival. No statistically significant difference was noted between the two treatment groups for the HR for overall mortality at maximum follow-up (HR 1.12, 95% CI 0.80, 1.56), disease-free survival (HR 0.97, 95% CI 0.74, 1.28), and loco-regional recurrence (HR 0.26, 95% CI 0.06, 1.18). Losing laryngeal functions was lower in concurrent chemoradiotherapy compared to sequential chemoradiotherapy (HR 0.28, 95% CI 0.13, 0.57).

### Preoperative radiotherapy versus postoperative radiotherapy

The RCT by Vanderbrouck et al. published in 1977 was the only trial comparing these two interventions [23]. This study involved 47 patients (preoperative radiotherapy: 24, postoperative radiotherapy: 23) with a mean follow-up of 5 years. The overall mortality was higher in patients receiving pre-operative radiotherapy compared to post-operative radiotherapy (HR 2.44, 95% CI 1.18, 5.03). There was no evidence of differences in treatment-related toxicity between the preoperative radiotherapy versus postoperative radiotherapy (HR 1.72, 95% CI 0.97, 3.03). The pattern of recurrence could not be assessed as this data was not available.

### Induction chemotherapy followed by concurrent chemoradiotherapy (IC-CRT) versus induction chemotherapy followed by radiotherapy (IC-RT)

We identified one trial by Yi et al. comparing these two interventions involving 113 patients (IC-CRT: 53, IC-RT: 60) with a mean follow-up of 3.4 years [24]. The primary outcomes available from this trial were overall survival, disease-free survival, and laryngectomy-free survival (LFS). There was no statistically significant differences in overall survival, laryngectomy-free survival and disease-free survival between the two treatment arms (overall survival: HR 0.98, 95% CI 0.72, 1.34; disease-free survival: HR 1.25, 95% CI 0.95, 1.64; LFS: HR 0.93, 95% CI 0.48, 1.82).

### Treatment-related toxicity

Of the five studies identified, quantitative analysis could only be performed for preoperative radiotherapy versus postoperative radiotherapy (one study [23]: HR 1.72, 95% CI 0.97, 3.03). In the remaining comparisons, only a narrative summary was possible and has been provided in **Table 2**. In the organ preservation versus non-organ preservation subgroup, the trial by Lefebvre et al. reported the number of toxicities severe enough to cause treatment interruptions [21], while the trial by Beauvillain et al. provided toxicity data following neoadjuvant chemotherapy only [20]. Similarly, for the concurrent versus sequential chemoradiotherapy subgroup, Prades et al. did not provide complication details following surgery [22]. Though effect estimates could not be calculated for the IC-CRT versus IC-RT comparison due to insufficient data, the trial by Yi et al. demonstrated an increased incidence of toxicity following IC-CRT compared to IC-RT (40.78% vs 5.08%, p<0.001) [24].

### Response-rates following neoadjuvant treatment

Descriptive analysis (**Table 3**) was performed for this outcome as none of the studies provided outcomes in the format that allowed quantitative analysis. In the non-organ preservation versus organ preservation comparison, response criteria were objectively defined by Lefebvre et al. while this information was unavailable in the trial by Beauvillain et al [20, 21]. Following 2-drug (cisplatin and 5-fluorouracil) neoadjuvant chemotherapy, Lefebvre et al. demonstrated 53.6% complete response rate [21]. However, Beauvillain et al. revealed a 14.4% complete response rate following induction chemotherapy [20].

### Sample size estimation

For trials reporting mortality as a time-to-event outcome, prior data indicated that the median survival time for surgically treated patients was 25 months [9]. If the true median survival time for the non-surgically treated patients is 13 months, we will need to recruit 43 participants in each arm over an accrual interval of 36 months and an additional follow-up period of 36 months, to be able to reject the null hypothesis as stated above with a power of 0.8 and Type I error probability of 0.05.

The summary of findings table is available in **S2 Table**.

## Discussion

### Summary of results

We included 5 RCTs which recruited 515 participants with resectable hypopharyngeal cancer and followed them over a mean follow-up period of 3.4 to 10.5 years. The comparison between non-organ preservation versus organ preservation had the maximum number of participants in this meta-analysis (284 participants) from two RCTs [20, 21]. However, no statistically significant difference could be demonstrated between the two interventions for overall survival, disease-free survival, recurrence-free survival (any recurrence, local, loco-regional and distal) after a follow-up period of 7.7 to 10.5 years.

The rest of the comparisons involved single RCTs. For the comparison between concurrent and sequential chemoradiotherapy (n = 71, mean follow-up 2 years), the hazard ratio revealed the superiority of concurrent chemoradiotherapy over sequential chemoradiotherapy in terms of laryngectomy-free survival (HR 0.28, 95% CI 0.13, 0.57) [22]. There were no significant differences between the two groups in terms of overall survival, disease-free survival, and loco-regional recurrence.

The comparison between pre- and post-operative radiotherapy was the only analysis where a statistically significant difference in terms of overall survival could be demonstrated [23]. The hazard ratio for death favoured surgery followed by post-operative radiotherapy (HR of preoperative vs post-operative radiotherapy 2.24, 95% CI 1.18, 5.03). However, the certainty of evidence was very low. No statistically significant difference was seen between the two interventions in terms of treatment-related toxicity.

The only comparison which employed contemporary chemotherapy regime (3-drug neoadjuvant chemotherapy) was the comparison between IC-CRT (induction chemotherapy followed by concurrent chemoradiotherapy) and IC-RT (induction chemotherapy followed by radiotherapy) [24]. No statistically significant difference was noted between the two intervention groups for overall survival, disease-free survival and laryngectomy-free survival (very low certainty of evidence).

### Applicability of the results

Current clinical practice for organ preservation techniques in hypopharyngeal cancers includes a 3-drug regime (taxane-based) and 3-dimensional conformal radiotherapy (intensity-modulated radiotherapy-IMRT). The 3-drug regime has been adopted into contemporary practice for hypopharyngeal cancer management based on trials conducted on laryngeal or other head and neck subsites with poor representation of hypopharyngeal cancers [25, 26]. Similarly, conformal radiotherapy is routinely employed in place of 2-dimensional planning without direct evidence from trials conducted on hypopharyngeal cancers [27]. All the studies included in this review were conducted in the pre-taxane and pre-IMRT era, except the trial by Yi et al. [24]. This highlights the need for future trials using current clinical practice, even if this current practice is not based on evidence from hypopharyngeal cancers.

The study by Yi et al. reported an increased incidence of toxicity following IC-CRT (induction chemotherapy followed by concurrent chemoradiotherapy) compared to IC-RT (induction chemotherapy followed by radiotherapy) (40.78% Vs 5.08%, p<0.001) [24]. These results reflect the outcomes of other landmark publications exploring the utility of IC-CRT for different head and neck subsites including a limited number of hypopharyngeal cancers (DECIDE and PARADIGM) [28, 29].

Though this study intended to identify the best treatment option for resectable hypopharyngeal cancers by meta-analytical methods, majority of the patients were categorized as T3 originating from the pyriform sinus (Table 1). These results should, therefore, not be extrapolated to T4 tumors and tumors originating from other hypopharyngeal subsites.

### Certainty of evidence

The overall certainty of evidence was low or very-low for all the comparisons (S2 Table). Except for the Lefebvre trial, in all the other included trials the randomization procedure was unclear [21]. Further, the outcome assessment blinding was not performed in any of the trials. Though survival outcomes would not be affected by blinding, lack of blinding can bias the estimates of other outcomes [30]. The sample sizes in the trials were small with fewer than 300 events for all comparisons and outcomes. The confidence intervals were wide for most outcomes. As a result, there was imprecision. These reasons led to downgrading the certainty of evidence.

### Strength and limitations

This is the first systematic review and meta-analysis on resectable hypopharyngeal squamous cell carcinoma to include evidence from randomised controlled trials only.

The search criteria did not include language restriction and were not restricted to published articles. We performed a thorough search for literature including a search of clinical trial registers. We used two independent researchers to select the studies and extract data to minimise the errors. We performed the analysis as per the Cochrane Handbook guidance and used the GRADE guidelines for risk of bias assessment.

Nevertheless, the evidence from the study is fraught with many limitations. As mentioned above, the certainty of evidence from our systematic review and meta-analysis is low or very low for all comparisons. We were unable to perform planned subgroup analyses because of paucity of data.

One of the objectives of this study was to assess the health-related quality of life and laryngectomy-free survival for various treatment options available for resectable hypopharyngeal cancers. None of the trials included in this meta-analysis included health-related quality of life metrics in their outcomes. There was also a paucity of data related to structured reporting of treatment-related toxicity in the trials included.

Despite the above limitations, this systematic review and meta-analysis on RCTs remains the best current evidence for the management of hypopharyngeal cancers.

### Agreement and disagreement with other systematic reviews and meta-analysis

Meta-analysis of chemotherapy in head and neck cancer (MACH-NC) published an update in 2011 to assess the impact of chemotherapy on various head and neck subsites [31]. This study demonstrated an absolute benefit in terms of survival to be 4.5% at 5-years for all subsites with the addition of chemotherapy. This was found to be 6.5% when chemotherapy was administered in the concurrent setting. In the subsite analysis, 66 comparisons were found to have been conducted on hypopharyngeal subsites involving 2767 patients. Similar to the findings of our study, no significant differences could be identified with the sequence of chemotherapy administration for hypopharyngeal subsites for various survival outcomes. MACH-NC, however, was not restricted to resectable hypopharyngeal cancers, unlike this meta-analysis. The latest iteration of MACH-NC published in 2021 however revealed that survival benefit was demonstrated only with concurrent chemotherapy but not with chemotherapy used in induction or adjuvant setting [32]. This update was nonetheless for all head and neck subsites.

Our literature review identified two systematic reviews and meta-analyses exclusively on resectable locally advanced hypopharyngeal cancers and one network meta-analysis on locally advanced laryngeal and hypopharyngeal cancers [2, 13, 33]. The reviews by Habib et al. and Cui et al. included randomized controlled trials as well as retrospective studies [13, 33]. Habib et al. reported that there was no significant survival difference between organ preservation and non-organ preservation strategy in terms of overall survival which is in agreement with our results [13]. Similar outcomes related to overall survival were noted in the meta-analysis by Cui et al [33]. However, Cui et al reported that the disease-free survival showed better outcomes with surgery compared to non-surgical modalities (risk ratio 1.20, 95% CI 1.03, 1.37) [33]. However, this comparison included retrospective studies along with randomized trials, which could explain the differences in conclusions between their study and ours. Trial sequential analysis performed in this meta-analysis proved inconclusive, thereby implying the need for future trials addressing the query about the best treatment option for locally advanced resectable hypopharyngeal cancers [33]. The network meta-analysis by Chen et al. (larynx and hypopharyngeal subsites) revealed a greater surface under curve ranking area (SUCRA) for surgery followed by radiotherapy compared to other treatment modalities in terms of three and five-year disease-free survival and overall survival [2]. As with the other meta-analysis, this study included non-randomised studies, which could explain the differences in conclusions between their study and ours.

### Future directions

There is a need for large, well-designed, multi-centric RCT with a low risk of bias in the future to support the best intervention for resectable hypopharyngeal cancers and to provide more credible guidelines. By improved patient selection, advances in delivering conformal radiotherapy, and newer chemotherapy drug combinations including targeted and immune-checkpoint inhibitors we will probably have different outcomes from those patients treated more than a decade ago. In a recent search on clinical trial registries, there are several randomized controlled trials (**Table 4**) focusing on combination therapies, which are underway and may provide future insights on the desired clinical outcomes. However, we were unable to identify trials focussing on health-related quality of life. Also, for any organ preservation trial in the larynx or hypopharynx, one must include laryngo-esophageal dysfunction-free survival as one of the main outcomes in the future [34].

**Table 4:** On-going randomized controlled trials evaluating treatment strategies for resectable locally advanced hypopharyngeal squamous cell carcinoma.

Trial Registry Details	Population	Intervention	Comparison	Outcome
Jprn-Umin (2008). "Randomized phase II syudy of weekly docetaxel versus S-1 and concurrent radiotherapy for stage III and IV laryngeal, oropharyngeal, and hypopharyngeal cancer in elderly patients or patients with complications	1) Histologically or cytologically confirmed squamous cell carcinoma 2) stage 3 or 4 with no evidence of distant metastases 3) resectable squamous cell carcinoma	Docetaxel with concurrent radiotherapy. A total radiotherapy dose of 70 Gy is planned with conventional fractionation (1.8–2 Gy/day). After a total dose of 40 Gy, patients with a 50% or greater decrease in the product of 2 perpendicular diameters of primary and neck tumors (responders) continued chemotherapy and completed radiotherapy. For non-responders with resectable tumor, definitive surgery was recommended. 4–6 weeks after the end of treatment	Administration of 2 courses (6 weeks) concurrent chemotherapy in which one course consists of S-1 65mg/m2 for 2 weeks administration followed by 1 week rest. A total radiotherapy dose of 70 Gy is planned with conventional fractionation (1.8-2Gy/day). After a total dose of 40 Gy, patients with a 50% or greater decrease in the product of 2 perpendicular diameters of primary and neck tumors (responders) continued chemotherapy and completed radiotherapy. For non-responders with resectable tumor, definitive surgery was recommended	Response rate, locoregional relapse free survival, survival with primary organ preservation, overall survival, treatment completion rate, incidence and severity of adverse events, economic analysis
ChiCtr (2018). "Clinical study of hypofractionated intensity-modulated radiotherapy for locally advanced hypopharyngeal carcinoma patients with no response to induction chemotherapy	Histologically proven malignant tumor of hypopharynx; stage III-IVb	Hypofractionated intensity-modulated radiotherapy	Conventional fractionated intensity-modulated radiotherapy	Local regional failure free survival, progression-free-survival, overall survival, toxicity
ChiCtr (2019). "Multicenter prospective clinical study for treatment options for partial release in patients with locally advanced laryngeal and hypopharyngeal cancer after TPF-induction chemotherapy." (TPF: docetaxel, cisplatin and fluorouracil)	Patients with advanced or advanced throat cancer (local tumor T2-4, any N) who achieved partial response after induction chemotherapy in 2–3 cycles	Surgery+ postoperative adjuvant radiotherapy	Radical radiotherapy or concurrent chemoradiotherapy	Overall survival, progression free survival, laryngeal preservation survival
ChiCtr-Iir (2015). "Nimotuzumab (Taixinsheng) combined with radiotherapy and chemotherapy for protecting the laryngeal function of locally advanced hypopharyngeal and laryngeal cancer patients: a multi-center, single-blind, randomized, controlled, phase II study."	Patients have no previous malignancy and a histologically proven squamous cell carcinoma of the stage III-IV larynx or hypopharynx	Nimotuzumab (200mg) combined with radiotherapy and cisplatin	Nimotuzumab (200mg) combined with radiotherapy and docetaxel	Laryngeal preservation, safety, local control survival, disease-free survival, overall survival
ChiCtr-Inr (2016). "A prospective, randomized trial of precision Neo-adjuvant therapy in hypopharyngeal carcinoma patients."	Hypopharyngeal squamous cell carcinoma	Adjuvant chemotherapy, Surgery and radiotherapy	Surgery and radiotherapy	Disease free survival, objective response rate, rate of endoscopic surgery, pathological complete response, local control rate, overall survival rate
ChiCTR2000036734 (2020). A comparative study between surgery and concurrent radiotherapy and chemotherapy in PR patients with advanced pharyngeal and laryngeal cancer. (PR: partial response)	Locally advanced (stage III, IV) laryngeal and hypopharyngeal carcinoma	Concurrent chemotherapy and radiotherapy	Surgery	Disease-free survival, overall survival, progression-free survival, disease control, laryngeal preservation rate, incidence of adverse effect, quality of life
NCT04502641. A Randomized Phase III Comparing Sequential Therapy With Induction Chemotherapy/Chemoradiotherapy To Cisplatinum-Based Chemoradiotherapy in Locally Advanced Hypopharyngeal Carcinoma	Hypopharyngeal cancers	Patients receive induction chemotherapy with docetaxel-based, with or without cisplatin or fluorouracil. Treatment repeats every 21 days for 3 courses. Then, patients receive cisplatin on day 1 day 21, 3 weeks as one cycle and undergo concurrent radiotherapy once daily, 5 days a week, for 6 to 7 weeks	Patients receive cisplatin on day 1 day 21, 3 weeks as one cycle and undergo concurrent radiotherapy once daily, 5 days a week, for 6 to 7 weeks.	Progression-free survival, overall-survival, adverse events rate

Abbreviations: T = tumor status; N = nodal status

## Conclusion

Of the various treatment combinations for resectable hypopharyngeal cancer compared, this study demonstrated a difference in survival only for the comparison between preoperative radiotherapy and postoperative radiotherapy (postoperative radiotherapy results in better outcome). Concurrent chemoradiotherapy was found to provide a better chance of preservation of the larynx compared to sequential chemoradiotherapy. The result of this meta-analysis indicates a lacuna in the currently available literature in terms of the best treatment modality for hypopharyngeal cancers. There is an imminent need to embark on an adequately powered trial with surgical and non-surgical arms.

### Differences between protocol and review

We originally planned to perform a network meta-analysis of the different interventions. However, because of the different types of participants included in different comparisons, the transitivity assumption was not met. Therefore, we performed only direct comparisons. The changes in methodology reflect these changes.

## Supporting information

S1 Table Search strategy. (DOCX)

S2 Table Summary of findings. (DOCX)

S1 Fig PRISMA checklist. (TIF)
