# Supplementary material for: Treatment options for resectable hypopharyngeal squamous cell carcinoma: A systematic review and meta-analysis of randomized controlled trials
Source: PLoS One. 2022 Nov 29;17(11):e0277460. doi: 10.1371/journal.pone.0277460 (PMC9707785; doi:10.1371/journal.pone.0277460)
Supplement: S1 Table — (DOCX) [file pone.0277460.s001.docx]

Table S1: Search Strategy

Medline

1. exp Hypopharyngeal Neoplasms/

2. exp Hypopharynx/

3. (hypopharyn* or laryngopharyn* or pharyngolaryn*).ab,ti.

4. 2 or 3

5. exp Neoplasms/

6. (carcinom* or cancer* or neoplas* or tumor* or tumour* or malignan* or SCC).ab,ti.

7. 5 or 6

8. 4 and 7

9. 1 or 8

10. chemoradiotherap*.ti,ab.

11. (surgery or surgical or excision or excisions or excise or resect* or (neck adj1 dissection*)).ti,ab.

12. (radiotherap* or brachytherap* or teletherap* or radiat* or irradiat*).ti,ab.

13. (chemotherap* or adjuvant or neoadjuvant or neo-adjuvant).ti,ab.

14. exp Radiotherapy/

15. exp Antineoplastic Agents/

16. exp surgical procedures, operative/ or lymph node excision/

17. exp Antimetabolites/

18. exp combined modality therapy/

19. or/10-18

20. 9 and 19

21. randomized controlled trial.pt.

22. controlled clinical trial.pt.

23. randomized.ab.

24. placebo.ab.

25. drug therapy.fs.

26. randomly.ab.

27. trial.ab.

28. groups.ab.

29. 21 or 22 or 23 or 24 or 25 or 26 or 27 or 28

30. exp animals/ not humans.sh.

31. 29 not 30

32. 20 and 31

Embase

1. exp hypopharynx cancer/ or exp hypopharynx carcinoma/ or exp hypopharynx squamous cell carcinoma/

2. exp hypopharynx/

3. (hypopharyn* or laryngopharyn* or pharyngolaryn*).ab,ti.

4. 2 or 3

5. exp malignant neoplasm/

6. (carcinom* or cancer* or neoplas* or tumor* or tumour* or malignan* or SCC).ab,ti.

7. 5 or 6

8. 4 and 7

9. 1 or 8

10. chemoradiotherap*.ti,ab.

11. (surgery or surgical or excision or excisions or excise or resect* or (neck adj1 dissection*)).ti,ab.

12. (radiotherap* or brachytherap* or teletherap* or radiat* or irradiat*).ti,ab.

13. (chemotherap* or adjuvant or neoadjuvant or neo-adjuvant).ti,ab.

14. exp radiotherapy/

15. exp cancer chemotherapy/

16. surgery/ or exp "head and neck surgery"/

17. or/10-16

18. 9 and 17

19. exp crossover-procedure/ or exp double-blind procedure/ or exp randomized controlled trial/ or single-blind procedure/

20. (((((random* or factorial* or crossover* or cross over* or cross-over* or placebo* or double*) adj blind*) or single*) adj blind*) or assign* or allocat* or volunteer*).af.

21. 19 or 20

22. 18 and 21

Cochrane

#1 MeSH descriptor: [Hypopharyngeal Neoplasms] explode all trees

#2 MeSH descriptor: [Hypopharynx] explode all trees

#3 (hypopharyn* or laryngopharyn* or pharyngolaryn*)

#4 #2 or #3

#5 MeSH descriptor: [Neoplasms] explode all trees

#6 (carcinom* or cancer* or neoplas* or tumor* or tumour* or malignan* or SCC):ti OR (carcinom* or cancer* or neoplas* or tumor* or tumour* or malignan* or SCC):ab

#7 #5 or #6

#8 #4 and #7

#9 #1 or #8

#10 chemoradiotherap*

#11 (surgery or surgical or excision or excisions or excise or resect* or (neck near/1 dissection*))

#12 (radiotherap* or brachytherap* or teletherap* or radiat* or irradiat*)

#13 (chemotherap* or adjuvant or neoadjuvant or neo-adjuvant)

#14 MeSH descriptor: [Radiotherapy] explode all trees

#15 MeSH descriptor: [Antineoplastic Agents] explode all trees

#16 MeSH descriptor: [Surgical Procedures, Operative] explode all trees

#17 MeSH descriptor: [Lymph Node Excision] explode all trees

#18 MeSH descriptor: [Antimetabolites] explode all trees

#19 MeSH descriptor: [Combined Modality Therapy] explode all trees

#20 #10 or #11 or #12 or #13 or #14 or #15 or #16 or #17 or #18 or #19

#21 #9 and #20

Science Citation Index and Conference Proceedings (Web of Science)

#1 TS=(hypopharyn* or laryngopharyn* or pharyngolaryn*)

#2 TS=(surgery or surgical or excision or excisions or excise or resect* or (neck near/1 dissection*) or radiotherap* or brachytherap* or teletherap* or radiat* or irradiat* or chemotherap* or adjuvant or neoadjuvant or neo-adjuvant)

#3 TS=(random* OR rct* OR crossover OR masked OR blind* OR placebo* OR meta-analysis OR systematic review* OR meta-analys*)

#4 #3 AND #2 AND #1

WHO ICTRP

Hypopharyngeal Cancer OR hypopharynx Cancer OR Laryngeal Cancer OR Larynx Cancer

ClinicalTrials.gov

Interventional Studies | Hypopharyngeal Cancer OR hypopharynx Cancer| Phase 2, 3, 4
