# Supplementary material for: Treatment options for resectable hypopharyngeal squamous cell carcinoma: A systematic review and meta-analysis of randomized controlled trials
Source: PLoS One. 2022 Nov 29;17(11):e0277460. doi: 10.1371/journal.pone.0277460 (PMC9707785; doi:10.1371/journal.pone.0277460)
Supplement: S2 Table — (DOCX) [file pone.0277460.s002.docx]

Table S3: Summary of Findings

| **Patient or population**: Resectable hypopharyngeal squamous cell carcinoma  **Setting**: Tertiary care centre | | | | | |
| --- | --- | --- | --- | --- | --- |
| Outcomes | **Anticipated absolute effects^*^** (95% CI) | | Relative effect (95% CI) | № of participants  (studies) | Certainty of the evidence (GRADE) |
|  | **Risk with Organ Preservation** | **Risk with Non-organ Preservation** |  |  |  |
| **Non-organ preservation compared to organ preservation** | | | | | |
| Over-all survival | 840 per 1,000 | **797 per 1,000** (593 to 942) | **HR 0.87** (0.49 to 1.55) [Over-all survival] | 284 (2 RCTs) | ⨁◯◯◯ VERY LOW ^a,b,c^ |
| Disease Free Survival | 880 per 1,000 | **926 per 1,000** (858 to 970) | **HR 1.23** (0.92 to 1.66) [Disease Free Survival] | 194 (1 RCT) | ⨁◯◯◯ VERY LOW ^a,c^ |
| Any Recurrence | 542 per 1,000 | **488 per 1,000** (276 to 851) | **RR 0.90** (0.51 to 1.57) [Any Recurrence] | 284 (2 RCTs) | ⨁◯◯◯ VERY LOW ^a,c^ |
| Local Recurrence | 80 per 1,000 | **85 per 1,000** (34 to 218) | **RR 1.06** (0.42 to 2.72) [Local Recurrence] | 194 (1 RCT) | ⨁◯◯◯ VERY LOW ^a,c^ |
| Loco-regional recurrence | 410 per 1,000 | **283 per 1,000** (152 to 525) | **RR 0.69** (0.37 to 1.28) [Loco-regional recurrence] | 284 (2 RCTs) | ⨁◯◯◯ VERY LOW ^a,c^ |
| Distal Recurrence | 285 per 1,000 | **368 per 1,000** (148 to 912) | **RR 1.29** (0.52 to 3.20) [Distal Recurrence] | 284 (2 RCTs) | ⨁◯◯◯ VERY LOW ^a,c^ |
| **Concurrent chemoradiotherapy compared to sequential chemotherapy followed by radiotherapy for resectable hypopharyngeal squamous cell carcinoma** | | | | | |
| Over-all survival | 490 per 1,000 | **530 per 1,000** (416 to 650) | **HR 1.12** (0.80 to 1.56) [Over-all survival] | 71 (1 RCT) | ⨁◯◯◯ VERY LOW ^a,c^ |
| Disease-free survival | 620 per 1,000 | **609 per 1,000** (511 to 710) | **HR 0.97** (0.74 to 1.28) [Disease-free survival] | 71 (1 RCT) | ⨁◯◯◯ VERY LOW ^a,c^ |
| Loco-regional recurrence | 206 per 1,000 | **54 per 1,000** (12 to 243) | **RR 0.26** (0.06 to 1.18) [Loco-regional recurrence] | 71 (1 RCT) | ⨁◯◯◯ VERY LOW ^a,c^ |
| Laryngectomy-free survival | 324 per 1,000 | **104 per 1,000** (50 to 200) | **HR 0.28** (0.13 to 0.57) [Laryngectomy-free survival] | 71 (1 RCT) | ⨁⨁◯◯ LOW ^a,d^ |
| **Preoperative radiotherapy compared to postoperative radiotherapy for resectable hypopharyngeal squamous cell carcinoma** | | | | | |
| Overall survival | 522 per 1,000 | **835 per 1,000** (581 to 976) | **HR 2.44** (1.18 to 5.03) [Overall survival] | 47 (1 RCT) | ⨁◯◯◯ VERY LOW ^a,c^ |
| Treatment-related toxicity | 609 per 1,000 | **1047 per 1,000** (591 to 1,845) | **Rate ratio 1.72** (0.97 to 3.03) | 47 (1 RCT) | ⨁◯◯◯ VERY LOW ^a,c^ |
| **Induction chemotherapy followed by chemoradiotherapy compared to induction chemotherapy followed by radiotherapy for resectable hypopharyngeal squamous cell carcinoma** | | | | | |
| Overall survival | 449 per 1,000 | **442 per 1,000** (349 to 550) | **HR 0.98** (0.72 to 1.34) | 113 (1 RCT) | ⨁◯◯◯ VERY LOW ^a,c^ |
| Disease-free survival | 439 per 1,000 | **514 per 1,000** (423 to 612) | **HR 1.25** (0.95 to 1.64) | 113 (1 RCT) | ⨁◯◯◯ VERY LOW ^a,c^ |
| Laryngectomy-free survival | 146 per 1,000 | **137 per 1,000** (73 to 250) | **HR 0.93** (0.48 to 1.82) | 113 (1 RCT) | ⨁◯◯◯ VERY LOW ^a,c^ |
| ***The risk in the intervention group** (and its 95% confidence interval) is based on the assumed risk in the comparison group and the **relative effect** of the intervention (and its 95% CI).   **CI:** Confidence interval; **HR:** Hazard Ratio; **RR:** Risk ratio | | | | | |
| **GRADE Working Group grades of evidence** **High certainty:** We are very confident that the true effect lies close to that of the estimate of the effect **Moderate certainty:** We are moderately confident in the effect estimate: The true effect is likely to be close to the estimate of the effect, but there is a possibility that it is substantially different **Low certainty:** Our confidence in the effect estimate is limited: The true effect may be substantially different from the estimate of the effect **Very low certainty:** We have very little confidence in the effect estimate: The true effect is likely to be substantially different from the estimate of effect | | | | | |
| Explanations   1. The risk of bias was unclear or high in the trials (downgraded by one level). 2. There was heterogeneity in the results between the trials (downgraded by one level) 3. The sample size was small, and the confidence intervals were wide (downgraded by two levels). 4. The sample size was small (downgraded by one level) | | | | | |
