# Supplementary figures and images for: Treatment options for resectable hypopharyngeal squamous cell carcinoma: A systematic review and meta-analysis of randomized controlled trials
Source: PLoS One. 2022 Nov 29;17(11):e0277460. doi: 10.1371/journal.pone.0277460 (PMC9707785; doi:10.1371/journal.pone.0277460)

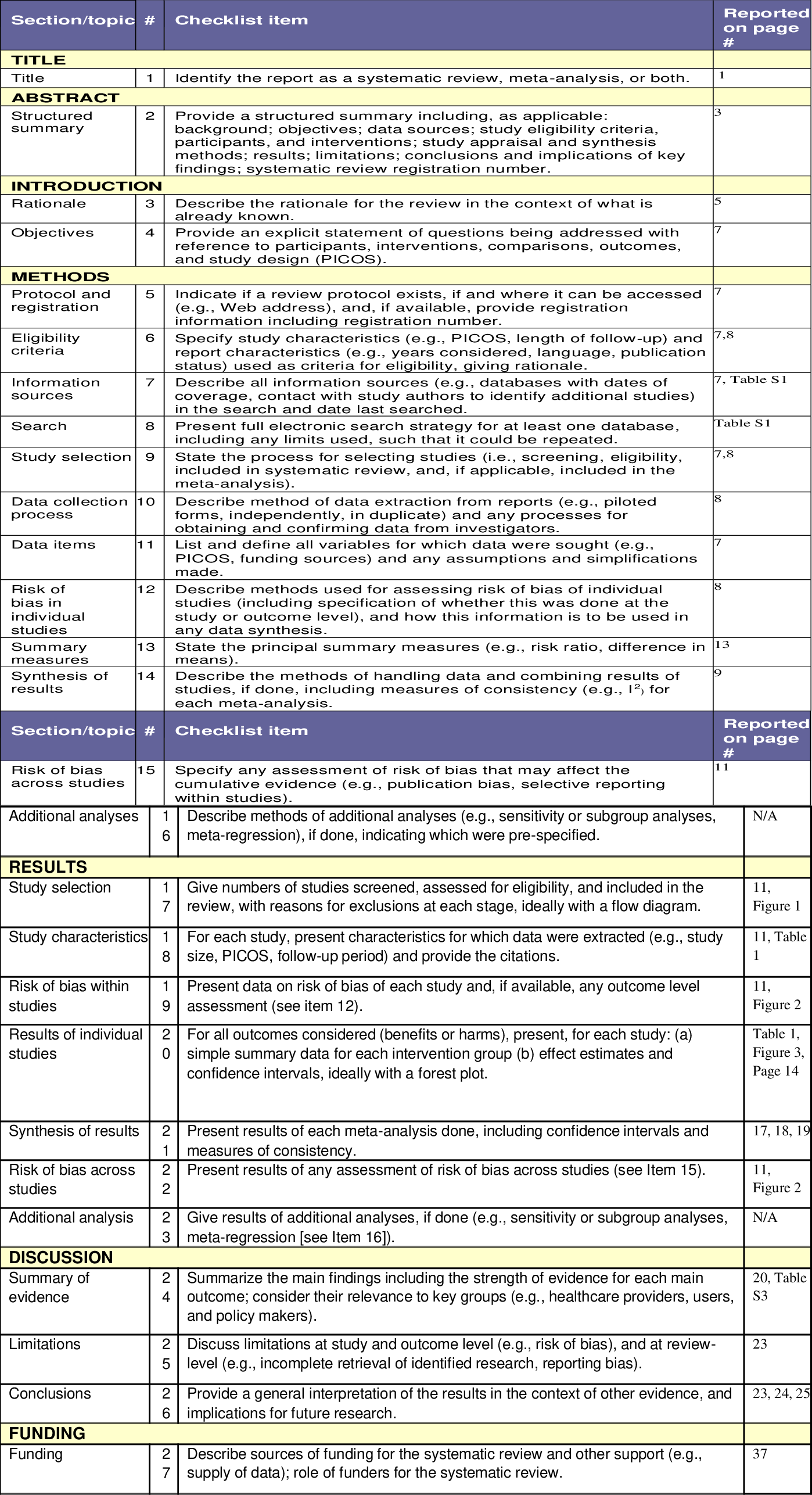

Supplement: S1 Fig — (TIF) [file pone.0277460.s003.tif]
